# Fitness costs of disrupting circadian rhythms in malaria parasites

**DOI:** 10.1098/rspb.2010.2457

**Published:** 2011-01-05

**Authors:** Aidan J. O'Donnell, Petra Schneider, Harriet G. McWatters, Sarah E. Reece

**Affiliations:** 1 Institute of Evolution, Institute of Immunology and Infection Research, University of Edinburgh, Edinburgh EH9 3JT, UK; 2 Centre for Immunity, Infection and Evolution, University of Edinburgh, Edinburgh EH9 3JT, UK; 3 Department of Plant Sciences, Oxford University, Oxford OX1 3RB, UK

**Keywords:** cell cycle, plasticity, periodicity, synchronicity, biological rhythms, *Plasmodium chabaudi*

## Abstract

Circadian biology assumes that biological rhythms maximize fitness by enabling organisms to coordinate with their environment. Despite circadian clocks being such a widespread phenomenon, demonstrating the fitness benefits of temporal coordination is challenging and such studies are rare. Here, we tested the consequences—for parasites—of being temporally mismatched to host circadian rhythms using the rodent malaria parasite, *Plasmodium chabaudi*. The cyclical nature of malaria infections is well known, as the cell cycles across parasite species last a multiple of approximately 24 h, but the evolutionary explanations for periodicity are poorly understood. We demonstrate that perturbation of parasite rhythms results in a twofold cost to the production of replicating and transmission stages. Thus, synchronization with host rhythms influences in-host survival and between-host transmission potential, revealing a role for circadian rhythms in the evolution of host–parasite interactions. More generally, our results provide a demonstration of the adaptive value of circadian rhythms and the utility of using an evolutionary framework to understand parasite traits.

## 1. Introduction

Circadian clocks underlie biological rhythms with a periodicity of approximately 24 h across a range of taxa, spanning from bacteria to plants, insects and vertebrates. All levels of biological organization within an organism, from gene expression to immune function, behaviour and seasonal patterns of reproduction are subject to regulation by the clock [1]. A cornerstone of chronobiology is the idea that organisms have evolved circadian clocks to allow coordination of physiology and behaviour with the Earth's daily rotation [2]. Despite circadian clocks being such a widespread phenomenon, demonstrating the fitness benefits of this coordination is challenging. Currently, the clearest evidence comes from experiments showing that having a circadian clock, the periodicity of which resonates with that of the environment, enhances the competitive ability of cyanobacteria [3] and plants [4], and larval growth rate in insects [5]. While considerable circumstantial evidence suggests that a circadian clock enhances fitness [6], studies unequivocally testing the adaptive significance of clocks are scarce for two reasons. First, the majority of recent research in the field of chronobiology has focused on asking questions about clock mechanisms [7–11]. Second, it is very difficult to do laboratory experiments that perturb timing schedules in ecologically realistic ways, and controlling for potentially confounding effects in field studies is equally challenging [12].

Here, we test whether matching developmental schedules to time of day affects the growth and transmission potential of malaria (*Plasmodium*) parasites. Malaria parasites replicate asexually in a vertebrate host and sexually in the mosquito vector. During the night, at the end of the cell cycle, each mature parasite (termed schizont) synchronously releases multiple daughter progeny (termed merozoites). *Plasmodium* species that infect humans have synchronous cell-cycle durations of 48 or 72 h and cause recurrent fever every 2 or 3 days, which is sufficiently precise to be a diagnostic feature of the disease [13]. Both the evolutionary and mechanistic explanations of this periodicity are poorly understood, but that it is always a multiple of 24 h suggests that circadian clocks regulate parasite rhythms. Every cell cycle, a proportion of parasites differentiate into male and female stages (gametocytes), which reproduce sexually when taken up by a mosquito. Rapid asexual replication is central to establishing and maintaining infections; the production of gametocytes is essential for transmission between hosts [14]. Malaria parasites offer a useful system for circadian studies because asexual and sexual stages can be distinguished and precisely quantified using molecular techniques developed specifically for this purpose [15–17]. Also, parasites are engaged in a life or death struggle with their hosts—so if perturbation of their cell cycle alters important interactions with their in-host environment, it will result in immediate and ecologically relevant fitness consequences.

There is increasing interest in the reciprocal approach of using unicellular taxa to test the generality of evolutionary theories developed for multicellular taxa and using an evolutionary approach to understand the biology of important unicellular taxa [14,18–20]. Matching the host circadian rhythm appears to be achieved using output from host clocks as a time cue for scheduling progression throughout the cell (replication) cycle. Previous work has demonstrated that if the rhythm of rodent malaria parasites is perturbed, it returns to match the host circadian rhythm within a few cell cycles [21–23]. Furthermore, human malaria parasites lose synchronicity in their cell cycle during *in vitro* culture [24], but the addition of melatonin appears to restore coordination [25]. Here, we show that perturbing the rhythm of parasites relative to the host body clock has consequences for their proliferation and transmission potential. Our study thus achieves a rare link between chronobiology and evolutionary biology, as well as representing a novel application of evolutionary theory to an organism of high medical importance.

## 2. Material and methods

### (a) Parasites and hosts

**Figure 1.**
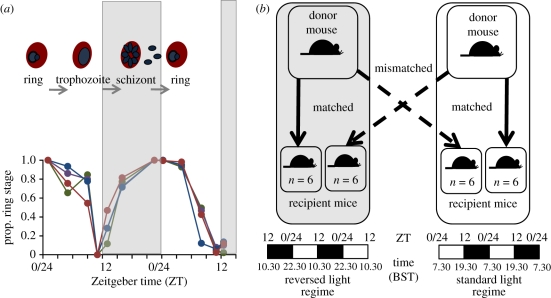
(*a*) The asexual cycle of *P. chabaudi* follows the pattern of day and night with ring stages being produced in the morning, which develop to trophozoites in the afternoon and release merozoites (progeny) from schizonts at night. Data are from four infections initiated and followed prior to our main experiment to verify that the *P. chabaudi* genotype used (AJ) has a synchronous and 24 h cell cycle. (*b*) The experiment was designed to test whether this temporal alignment is beneficial to parasite replication and transmission. Arrows indicate transfers of parasites to recipient hosts (four groups of six mice) within and between two rooms with different lighting schedules. Parasites remaining in the same room acted as controls as they were matched to host rhythms. Parasites transferred to hosts with a different rhythm from their donor were temporally mismatched, analogous to jetlag. Dark and light bars indicated lights-on/lights-off status throughout each 48 h period. Zeitgeber time (ZT) is displayed above the bars; ZT 0/24, time of lights-on and ZT 12, time of lights-off.

Hosts were 10–12-week-old MF1 male mice housed at 21°C with ad libitum food and drinking water supplemented with 0.05 per cent para-aminobenzoic acid (to supplement parasite growth). *Plasmodium chabaudi* has previously been reported to have a synchronous cell cycle of 24 h [26], but—prior to our main experiment—we set up infections to verify that this was also the case for the clone (AJ) used here. For this study, we initiated four replicate infections with 1 × 10^6^ parasitized red blood cells (RBCs) in mice maintained on a 12 L : 12 D cycle. We followed the proportion of parasites at ring stage at approximately four-hourly intervals over 36 h on days 3 and 4 post-infection (pi). These data are presented in figure 1*a* and demonstrate unambiguously that the cell-cycle clone AJ is synchronous with a duration of 24 h.

Our main experiment required manipulating the circadian rhythms of hosts. We achieved this by housing mice in two rooms, each maintained on a 12 L : 12 D cycle, that differed only in the timing of ‘lights-on’. In the ‘standard schedule’ room, lights were on during the day (lights-on: 07.30 h; lights-off: 19.30 h); in the ‘light-reversed’ room, lights were on during the night (lights-on 22.30 h; lights-off: 10.30 h). All mice in the experiment were allowed to acclimatize to their respective lighting regimes for two weeks before infection. This allowed mice time to entrain to their schedule, as previous work has demonstrated this occurs within 7 days [27]. However, prior to infecting the mice, we verified that they behaved as expected for their light : dark schedule, i.e. were active during the dark period and inactive when lights were on. In each room, a host was infected with 1 × 10^6^ *P. chabaudi* (clone AJ) parasitized RBCs to provide ‘donor’ parasites to initiate experimental infections (figure 1*b*). Mice used in the experiment were housed in groups of three and a total of 24 were used (*n* = 6 infections per treatment group).

### (b) Experimental design

Parasites at the ring stage from the donor infection in each room were used to infect hosts (with 1 × 10^6^ parasitized RBCs) in both rooms (figure 1*b*). Parasites originating from the ‘standard regime’ room were collected at Zeitgeber time (ZT) 0 (the time of lights-on) and used to infect *simultaneously* mice in the same room and the light-reversed room. The same procedure was repeated 15 h later, at ZT 0 in the light-reversed room for parasites originating from this room, which were again used to infect *simultaneously* mice in the light-reversed room and the standard regime room. This produced two treatments: parasites ‘matched’ to the host rhythm (control infections; mice infected with parasites from the same room) and parasites ‘mismatched’ to the host rhythm (experimental infections; mice infected with parasites from the room on the opposite lighting schedule). Parasites in the mismatched treatments thus underwent a temporal phase shift, analogous to the cross-continental travel that induces jet lag. This experimental design provides four cross-factored groups of infections (two schedules of origin × two destination schedules) and enables the performance of mismatched parasites' growth and transmission potential to be compared with those matched to host rhythms in both the original and the destination rooms.

### (c) Data collection

All mice were sampled twice daily, in the morning at 09.00 h and in the evening at 19.00 h (BST), during the growth phase of infections (that is, from days 0–7 pi [28]). Focusing on the growth phase minimized the influence of potentially confounding variables, such as anaemia and immune responses, which significantly influence parasitaemia after peak, and avoided the risk of host mortality, causing an unbalanced design and reducing power. At each sampling point, thin smears were made, samples were taken to quantify gametocyte (10 µl) and total parasite (5 µl) densities, and RBC densities measured using flow cytometry (Beckman Coulter; for 09.00 h samples only). Thin smears were made from tail blood and stained with 20 per cent Giemsa buffered in 80 per cent phosphate buffer solution for 20 min. These smears were used to determine the cell-cycle stage of parasites; 200 parasites per smear were examined and each classified as one of the following stages: ring, trophozoite, schizont or gametocyte [26]. In the few smears where the parasitaemia was very low, only 100 (or in very rare cases 50) parasites were examined. The densities of gametocytes and total parasites were measured using reverse transcriptase–quantitative PCR (RT–qPCR) and qPCR, respectively. Blood samples were taken for DNA (5 µl) and for RNA (10 µl); DNA and RNA were extracted using the ABI Prism 6100 and cDNA was obtained from RNA using the high-capacity cDNA archive kit (Applied Biosystems) according to the manufacturer's protocol [17]. Total parasite and gametocyte densities were obtained using primers based on the gametocyte-expressed gene PC302249.00.0 [29]. This protocol was applied to DNA to give a density of total parasites (qPCR) and also on cDNA to count gametocyte density (RT–qPCR) specifically. We counted total parasites from samples taken on days 1, 3, 5 and 7 and we counted gametocytes from all samples (days 1–7).

### (d) Statistical analysis

We used R v. 2.6.1 (The R Foundation for Statistical Computing, Vienna, Austria; http://www.R-project.org) for all analyses. To verify the validity of the methods we used, several checks were necessary: specifically, that (1) sequestration and (2) qPCR assays did not bias density estimates when parasites were sampled late in the cell cycle. For these analyses, we investigated whether: (1) parasite densities differed between early and late sampling points within the same cell cycle owing to sequestration of trophozoite and/or schizont stages and (2) parasite densities, estimated by qPCR and smear from the same set of samples, differed according to the method used. As these analyses involved paired data, we used one-sample *t*-tests to test whether the mean differences between pairs of (1) early/late samples and (2) qPCR/smear estimates were significantly different from zero. Having verified our methods, we then used general linear models to analyse our experimental data by testing whether: (3) timing of the parasite cell cycle of matched (control) infections differed between rooms; (4) parasites in the matched groups differed in performance across the rooms; and (5) there were consequences of being matched or mismatched to host rhythm for total parasite and gametocyte densities. The details of each analysis are explained below.

#### (i) Validation of experimental procedures

(1) *Sequestration during development*. We tested the possibility that sequestration of parasites in late stages of development (trophozoites and schizonts [30,31]) could bias estimates of parasite density and developmental stage using the matched (control) treatments. If late-stage parasites sequester, parasitaemia estimates from blood smears will appear to be lower in samples taken at the dark period late in the cell cycle compared with those taken during the light period when parasites are at ring or early trophozoite stages. There was no significant difference in the parasitaemia of each infection between subsequent sample points on day 3 (60–72 h pi for the light-reversed room and 72–84 h pi for the standard regime room; *t* = 2.05; *p* = 0.065), suggesting that sequestration does not significantly bias parasite estimates from samples taken later in the cell cycle.

(2) *Assays for parasite density*. We investigated the possibility that qPCR (which counts genomes) could overestimate parasite density in samples taken late in the cell cycle when mitotic division during maturation into schizonts may have begun. To test whether qPCR overestimates parasite density (i.e. the number of infected RBCs per millilitre) relative to estimates from blood smears, we examined whether the difference in density estimates from qPCR and blood smears changes throughout the cell cycle (i.e. do the estimates from qPCR increase throughout the cell cycle more than estimates from smears?). Specifically, we compared the change in densities between samples taken on day 3 pi early and late in the same cell cycle (i.e. 72 h pi for the light-reversed room and 84 h pi for the standard schedule room) for all infections and found no significant difference (*t* = 1.66; *p* = 0.11). Furthermore, we examined blood smears from each infection from days 3 to 7 to investigate whether schizonts were present in the circulation and, if so, whether their prevalence increased as infections progressed. The average number of schizonts observed each day, in approximately 3500 red blood cells, ranged from 1.5 to 2.1 and did not show any temporal trends or variation across treatment groups. Therefore, the very low prevalence of schizonts in blood smears suggests that the potential inaccuracies of qPCR (by falsely counting multiple genomes within a single schizont) are negligible.

#### (ii) Experiment

(3) *Schedule manipulations.* To verify the experimental manipulations had been successful at creating different parasite schedules in each room, the developmental stages of parasites in matched (control) infections in the standard schedule room were compared with matched (control) infections in the light-reversed room. We compared these parasite schedules at 60 h pi as this was the earliest sampling point when sufficient parasites could be detected for staging by microscopy.

(4) *Performance of matched infections*. To test whether the matched (control) groups differed in performance across the light-reversed and standard schedule rooms, we compared the overall performance of these groups. Specifically, we compared cumulative parasite densities produced by infections in each matched group. A significant difference between the two matched groups would suggest that a room-of-origin effect carried over into the experimental infections and invalidated comparisons between mismatched infections and matched infections in the same destination room.

(5) *Effects of mismatch to host circadian rhythm*. To investigate the effects of mismatch on the densities of total parasites and gametocytes, both the treatment (matched or mismatched) and the light regime (standard or light-reversed schedule) and their interaction were fitted and models simplified using stepwise deletion [32]. We used the cumulative densities of parasites or gametocytes, calculated for each infection from days 1 to 7 pi. As infections originating from the standard schedule room are ahead of infections originating from the light-reversed room, the same *duration of infection* occurs in the morning for infections from the standard schedule room and in the evening for infections from the light-reversed room. Therefore, for the analysis of total parasite densities, we have four sampling points for each treatment group, where the duration of infections is consistent because we counted parasites every other day from the initiation of the infections. These points occur at days 1, 3, 5 and 7 pi (durations of 24, 72, 120 and 168 h pi), being the samples collected at 09.00 h for parasites originating from the standard schedule room and at 19.00 h for parasites from the light-reversed room.

In contrast to counts of total parasites, gametocyte densities are several orders of magnitude lower. To maximize our power, we counted gametocytes from all sampling time points (days 1–7 pi; 12–168 h pi), and all samples contributed to the cumulative density for each infection. We also tested whether our match/mismatch treatment influenced how much variation in the synchronicity of cell-cycle schedules occurs within infections. We compared the coefficient of variation (standard deviation of the mean) for the proportion of ring-stage parasites observed in blood films throughout infections (days 3–7 pi; 12–168 h pi) in the matched and mismatched groups.

## 3. Results

### (a) Entrainment of parasite rhythms

Our ‘jetlag’ experimental design required that infections in the two rooms had different phases relative to each other. To verify that the phase of the cell-cycle rhythm was set by the lighting schedule, the developmental stage of parasites was examined when parasites in each treatment reached 60 hpi. At 60 hpi in the light-reversed room, lights were on and parasites were expected to be at the ring stage, whereas in the standard schedule room, lights were off and so parasites should be at later cell-cycle stages. As expected, a greater proportion of ring-stage parasites was observed in infections originating from the light-reversed room than the standard schedule room (*F*_1,17_ = 12.29; *p* = 0.003), demonstrating that parasites originating from each room had differently phased cell-cycle rhythms.

We also tested whether there was a significant difference in the performance of infections in the two matched (control) groups, which would suggest that a room-of-origin effect carried over into the experimental infections. However, the cumulative parasite densities of infections in these groups did not differ significantly (*F*_1,10_ = 1.40; *p* = 0.265), revealing that the matched infections performed similarly, regardless of their lighting schedule. This enables the performance of mismatched parasites to be compared with both groups of matched infections (i.e. to matched infections in the same destination room, as well as matched infections remaining in the room of origin).

### (b) Effects of mismatch to host circadian rhythm

**Figure 2.**
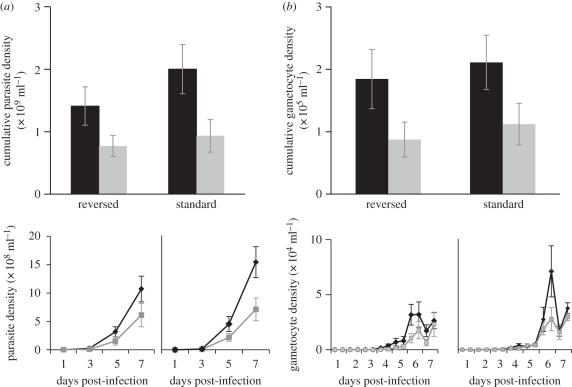
(*a*) Total and (*b*) gametocyte (sexual) densities of infections initiated with parasites matched or mismatched to their host rhythm, in standard and reversed light schedules, followed from (*a*) 24 h or (*b*) 12 h p.i. to 168 h. The top panels show the mean cumulative (±s.e.) densities achieved by infections in hosts (*n* = 6 per group) on the same (matched, black bars) or opposite (mismatched, grey bars) schedule. In each graph, the left pair of bars represents infections originating from the light-reversed room and the right pair of bars represents infections originating from the standard schedule room. The lower panels decompose the cumulative densities into the temporal dynamics of infections in the treatment groups in each room: the mean (±s.e.) densities of matched (black lines) and mismatched (grey lines) infections are plotted. The left graph of each point represents infections originating from the light-reversed room and the right graph of each point represents infections originating from the standard schedule room. The difference in the number of points plotted in the lower panels (and contributing data to the top panels) is due to the lower frequency of analysing samples to count total parasites (*a*) than gametocytes (*b*).

There was a strong effect of perturbing parasite cell cycle relative to the host rhythm (figure 2). We found significant effects of our mismatch/match treatment on the production of total parasites and gametocytes, but neither the original schedule nor its interaction with treatment significantly influenced infections (table 1). The cumulative parasite densities (figure 2*a*, upper panel) of matched infections were double those of mismatched infections (*F*_1,22_ = 8.38; *p* = 0.008; matched mean = 1.7 ± 0.25 × 10^9^ ml^−1^; mismatched = 0.85 ± 0.15 × 10^9^ ml^−1^). Cumulative gametocyte densities (figure 2*b*, upper panel) followed the same pattern, in which mismatched infections produced significantly fewer gametocytes (*F*_1,22_ = 6.84; *p* = 0.016; matched mean = 1.9 ± 0.21 × 10^5^ gametocytes ml^−1^; mismatched = 1.01±0.21 × 10^5^ ml^−1^). The cumulative densities (upper panels) are decomposed into their temporal dynamics (lower panels) in figure 2. All *F*-ratios and *p*-values are given in table 1, along with the mean differences in the cumulative densities of mismatch and matched infections.

**Table 1.** The effects of schedule perturbation treatment (matched/mismatched), room (schedule) of origin and their interaction on the performance of infections. *F*-ratios and associated *p*-values for all terms are given along with the mean (±s.e.) difference (matched–mismatched) for significant effects and the adjusted *R*^2^ for minimal models.

	*F*-ratio	*p*-value	mean difference
parasite density (adj *R*^2^ = 0.24)			
treatment (matched/mismatched)	*F*_1,22_ = 8.38	0.008	8.54 ± 2.9 × 10^8^ ml^−1^
original schedule (reversed/standard)	*F*_1,21_ = 0.51	0.482	—
treatment × schedule	*F*_1,20_ = 1.64	0.215	—
gametocyte density (adj *R*^2^ = 0.20)			
treatment (matched/mismatched)	*F*_1,22_ = 6.84	0.016	9.80 ± 3.7 × 10^4^ ml^−1^
original schedule (reversed/standard)	*F*_1,21_ < 0.01	0.979	—
treatment × schedule	*F*_1,20_ = 0.44	0.516	—
coefficient of variation (adj *R*^2^ = 0.14)			
treatment (matched/mismatched)	*F*_1,22_ = 4.69	0.041	0.03 ± 0.015
original schedule (reversed/standard)	*F*_1,21_ = 3.35	0.082	—
treatment × schedule	*F*_1,20_ = 1.33	0.262	—

Previous studies suggest that, when perturbed, parasite rhythms change to re-align with the host rhythm. Therefore, we hypothesized that as parasites adjust to their new environment, the schedules of mismatched parasites should become increasingly different from those of matched infections remaining on the original schedule, and increasingly similar to the schedules of matched infections in the same destination room. This adjustment is predicted to cause greater variation between the schedules of mismatched and matched infections, and this is reflected by significantly greater coefficients of variation in mismatched than in matched parasites (*F*_1,22_ = 4.69; *p* = 0.041). Furthermore, neither the original schedule nor its interaction with treatment significantly influenced the extent of synchronicity in cell-cycle schedules (table 1).

## 4. Discussion

Our data provide a rare demonstration of the impact that circadian rhythms have on fitness. Specifically, we reveal that parasites forced out of synchrony with the host's schedule paid substantial costs, as a single phase shift reduced both in-host replication and the production of transmission stages by around 50 per cent. These costs are likely to have broad implications for parasite survival and reproduction. Malaria parasites must optimize the trade-off between investment in replication for in-host survival and the production of gametocytes for between-host transmission. Parasites with low replication rates are vulnerable to clearance by the immune system, anti-malarial drugs and are poor competitors in genetically mixed infections [33–37]. For example, subtle differences in the replication rate of co-infecting strains can lead to substantial competitive suppression in mixed infections [33,38,39]. Replication rate is also a key factor in determining the production of transmission stages [28]. For the range of gametocyte densities observed in our data, there is a strong positive relationship with mosquito infectivity [28,40–42] in terms of both the prevalence and intensity of mosquitoes infected. The greater variation in cell-cycle schedules in mismatched infections suggests that an interaction between the synchronicity and timing of cell-cycle rhythms shapes the dynamics of infections. More broadly, our data suggest that circadian rhythms play an important role in the evolution of host–parasite interactions. Much recent research on circadian clocks in a disease context focuses on the implications of infection for host rhythms [43–45]. However, our results complement observations that perturbation of host clocks shifts parasites' rhythms—across a variety of taxa [46–48]—and suggests that rhythms are an important but unappreciated selection pressure on parasites.

Understanding how parasites achieve their coordination will facilitate explaining why parasites synchronize cell cycles with host rhythms. A key question is whether the timing and synchronicity of the cell cycle of parasites is a plastic (actively adjusted) trait. The developmental schedule of an asexual parasite can be conceptually split into remodelling the red blood cell, feeding and, finally, replication. Whether there is plasticity in the duration of these processes is yet to be investigated, but it is possible that development time may trade off against the number of progeny produced since each nucleus within a maturing parasite can divide a different number of times [49]. Recent experiments showing that melatonin can speed up and synchronize the development of *Plasmodium falciparum* in culture suggest that parasites might use host melatonin as a time cue [25]. However, these experiments applied melatonin at substantially higher than physiological concentrations, and we have been unable to repeat these studies in our laboratory (S. E. Reece & H. G. McWatters 2009, unpublished data). If cell-cycle duration is plastic, it will be important to test whether development speeds up or slows down and identify the cues used to schedule development, as this may have implications for disease control. For example, if cell cycles can be slowed, quiescent parasite stages may reduce proliferation rate (reduce pathology), but may also be less sensitive to drug treatment (act as a resistance trait) [36,50–52].

Alternatively, parasite cell-cycle schedules and synchronicity might be passively maintained by host factors with a circadian basis. In this scenario, there may be sufficient variation in the cell-cycle duration of parasites within a cohort that, following perturbation, a proportion will be, by chance, on the correct schedule and so form the next cohort [53]. This could occur as a result of host responses to schizogony, such as fever or cytokine spikes, both of which may kill parasites that are on a slower schedule [54,55]. While fever may play a role in human malaria infections, mice do not experience fever at schizogony, which suggests that other host factors must be involved in synchronizing parasites. Whether synchronicity and circadian development are the results of an active parasite strategy, or a passive host effect, the speed at which parasite schedules recover from perturbation will depend on many factors, including the costs/benefits of mismatch, how much variation exists in the development time of each cohort, the duration of the ‘gate’ that selects which parasites contribute to the next cell cycle, and the accuracy with which parasites can detect and respond to time cues.

It is important to distinguish between explanations for circadian rhythms that merely require synchronicity and those that require synchronicity to be linked to environmental rhythms. For example, one explanation, discussed above, for parasite synchronicity is that ‘safety in numbers’ protects progeny when they are released into the blood stream at schizogony. However, this explanation only requires parasites to be coordinated with each other, not with the host. The Hawking hypothesis predicts that parasite cell cycles are timed so that the maturation of each cohort of gametocytes coincides with mosquito-biting activity [56,57], implying that vector rhythms are the relevant environmental parameter. However, data available across a range of *Plasmodium* species are not supportive. For instance, human malaria (*P. falciparum*) gametocytes do not show diurnal rhythms in infectivity to mosquitoes [58,59] and are infectious for at least 7 days [60,61]. Temporal coordination may benefit parasites in two ways: by facilitating exploitation of circadian-dependent host resources, such as the release of new red blood cells [62,63], or avoidance of interactions with host immune factors, such as TNF-α or IL-6, which are secreted with a circadian rhythm [43]. Interestingly, TNF-α is a major component of the immune response (paroxysm) initiated by the synchronous release of parasites at schizogony and can ‘sterilize’ gametocyte infectivity for several hours [64]. That cell cycles are timed to end at night, when vectors are active, suggests parasite rhythms are either the resolution of a significant trade-off or a serious constraint [14].

Evolutionary ecology is concerned with explaining variation and its fitness consequences. Studies asking the fundamental evolutionary question of why circadian clocks are important for an organism provide the necessary context for work focusing on circadian mechanisms [6]. Circadian clocks have evolved multiple times because many organisms are exposed to the daily changes in light and temperature resulting from the planet's rotation. Across taxa, there is little homology between clock proteins [65], but complex, interlocked feedback loops and close associations with light and/or temperature input pathways are features of all known clockworks [10,66]. The repeated observation of such mechanisms lends support to the idea that the clock's ultimate purpose is to track seasonal changes and couple endogenous timekeeping with environment rhythmicity. Chronobiology has historically been neglected by evolutionary ecologists, but this is changing as it offers a novel opportunity for a holistic approach: because the mechanics of circadian clocks are well known from multiple model systems (including fungi [8,67], insects [7] and mammals [9]), there are real opportunities to link mechanistic and evolutionary explanations for an important trait. However, there are substantial challenges associated with linking trait variation and underlying physiological mechanisms, not least the difficulty of assessing the effect of the clock on fitness in a context resembling that of the real world (most experimental designs have considered the effects of non-24 h light : dark cycles [3–5]). The wealth of cell and molecular biology data available for malaria parasites, and the ability to investigate and manipulate their traits *in vivo* and *in vitro*, offer a powerful means to set chronobiology within an evolutionary framework.
